# Supplementary material for: Laminar signal extraction over extended cortical areas by means of a spatial GLM
Source: PLoS One. 2019 Mar 27;14(3):e0212493. doi: 10.1371/journal.pone.0212493 (PMC6436691; doi:10.1371/journal.pone.0212493)
Supplement: S1 File — (PDF) [file pone.0212493.s001.pdf]

# Supporting Information

## Appendix A: Orientation dependent partial volume distribution

In order to find out how different laminae are distributed over voxels, it is important to know the orientation and location of the surface with respect to the voxels. Here we analytically describe an algorithm to solve this problem.

The intersection of a laminar surface and a voxel is approximated by the intersection of a cuboid and a plane that are arbitrarily positioned and oriented with respect to each other.

The voxel grid is given by three primitive lattice vectors $\{\vec{a_1}, \vec{a_2}, \vec{a_3}\}$, having the orientation and length of the voxel edges. The lattice vectors are usually but not necessarily oriented along the cardinal axes. For a cubic voxel grid with edge length $L$ the primitive lattice vectors are $\{L\hat{x}, L\hat{y}$ and $L\hat{z}\}$. A voxel can be indexed with three integers $m_i$. The centre position $\vec{m}$ of the voxel is

$$\vec{m} = \sum_{i=1}^{3} m_i \vec{a_i} \tag{1}$$

and the 8 corner positions $\vec{c}$ of the voxel are

$$\vec{c} = \sum_{i=1}^{3} \left( m_i \pm \frac{1}{2} \right) \vec{a_i}. \tag{2}$$

The voxel volume is $V = \vec{a_1} \cdot (\vec{a_2} \times \vec{a_3})$.

A plane can be defined by a vector $\vec{N}$. The plane is perpendicular to $\vec{N}$ and has distance $1/||\vec{N}||$ to the origin. The plane is given by all points $\vec{r}$ satisfying

$$\vec{r} \cdot \vec{N} = 1. \tag{3}$$

It is useful to express $\vec{N}$ in terms of reciprocal lattice vectors $\vec{b_i}$:

$$\vec{N} = \sum_{i=1}^{3} N_i \vec{b_i}. \tag{4}$$

The reciprocal basis vector $\vec{b_1}$ is defined as $\vec{b_1} = \vec{a_2} \times \vec{a_3}/V$, $\vec{b_2}$ and $\vec{b_3}$ are obtained by cyclic permutation. For a cubic voxel grid, the reciprocal basis vectors are $\vec{b_1} = \hat{x}/L$, $\vec{b_2} = \hat{y}/L$ and $\vec{b_3} = \hat{z}/L$. By construction $\vec{a_i}$ and $\vec{b_i}$ are orthonormal to each other

$$\vec{a_i} \cdot \vec{b_j} = \delta_{ij}. \tag{5}$$

Points on the four edges of voxel $\vec{m}$ in the direction $\vec{a_1}$ are of the form

$$\vec{r} = (m_1 + \lambda_1)\vec{a_1} + \sum_{i=2}^{3} (m_i \pm \frac{1}{2})\vec{a_i}, \quad |\lambda_1| \leq \frac{1}{2}. \tag{6}$$

In view of 4 the four possible intersection points of $\vec{N}$ with the edges parallel to $\vec{a_1}$ are given by the four $\lambda_1$ of the form

$$\lambda_1 N_1 = 1 - \sum_{i=1}^{3} m_i N_i \pm \frac{1}{2} N_2 \pm \frac{1}{2} N_3. \tag{7}$$

Analogously the possible intersection points with the edges parallel to $\vec{a_2}$ and $\vec{a_3}$ are given by four $\lambda_2$ and $\lambda_3$ values respectively. Of the 12 possible intersection points, those with $|\lambda_i| \leq \frac{1}{2}$ give the actual (at most 6) intersection points. The area of the polygon connecting the intersection points can be readily computed, as well as the volume on either side of the plane.

At this point we have expressions for the intersection point that are independent of the choice of the voxel edge vectors $\vec{a}_i$. Explicit expressions can be obtained for the intersection points and intersection area. To be explicit, consider the intersection of a plane, moving with a given orientation, i.e. $\vec{N} = t\vec{n}$ where $t$ takes arbitrary real values and $\vec{n}$ stays constant. A finite intersection is only found for the range of $t$ values between the maximum and minimum value of $1/[\sum_{i=1}^{3}(m_i \pm \frac{1}{2})n_i]$.

## Appendix B: Spring-Mass System

### Introduction

The cerebral cortex consists of a convoluted surface of gyri and sulci. There are accurate models for the gyrification of the cortex as a whole [2] and there is a description of how the layers within the cortex behave [1]: the volume ratio between layers is equal for an arbitrarily curved pieced of cerebral cortex. This is what has become known as the Bok-principle. Here we describe how to simulate data for a two dimensional system that obeys Bok's principle by means of a spring-mass system. A spring-mass system was chosen, because it is independent of the algorithms by means of which we estimate curvature in the volume. This makes it ideal benchmark data for the methods presented in the body of this paper.

### Spring-Mass System

The cortex is modelled by means of a spring mass system. This is an approximation of the cortex consisting of quadrilaterals. Each quadrilateral consists of four edges and four vertices, which are the springs and masses respectively. The collection of springs and masses that form quadrilaterals will henceforth be referred to as the system.

The system has total energy $U$. In the present simulation only a single contribution to the energy is considered, related to the area of each quadrilateral. More generally, other contributions could be taken into account, for example relating to the length of each edge:

$$U = U_{area} + U_{edge} + ... \tag{8}$$

We are looking for the case where the energy is minimised, as this is when the system has come to rest and all quadrilaterals have reached an equilibrium area. Note that only the vertices are displaced in the first instance; the edges and quadrilaterals are formed as a consequence. The energy decreases by moving the vertices in the direction of the net force applied to them. The force $\vec{F}_n$ on vertex $n$ is minus the derivative of $U$ with respect to the position $\vec{r}_n$ of that vertex:

$$\vec{F}_n = -\nabla_n U. \tag{9}$$

A quadrilateral $Q_i$ is defined to be the space enclosed by four vertices in two dimensions $\vec{u}_i$, $\vec{v}_i$, $\vec{w}_i$ and $\vec{k}_i$, shown in Fig S1.

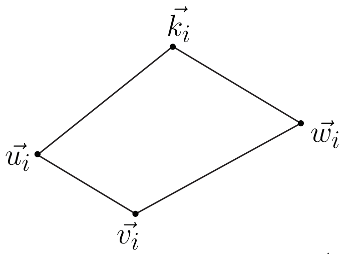


**Fig S1.** Quadrilateral $Q_i$, consisting of vertices $\vec{u}_i$, $\vec{v}_i$, $\vec{w}_i$ and $\vec{k}_i$.

Its area $A_i$ is a scalar function depending on the vertices of the quadrilateral, $A_i = A_i(\vec{u}_i, \vec{v}_i, \vec{w}_i, \vec{k}_i)$:

$$A_i = \frac{1}{2}[\left(u_{i,y}v_{i,x} - u_{i,x}v_{i,y}\right) + \left(v_{i,y}w_{i,x} - v_{i,x}w_{i,y}\right) + \ldots \quad (10)$$

$$\left(w_{i,y}k_{i,x} - w_{i,x}k_{i,y}\right) + \left(k_{i,y}u_{i,x} - k_{i,x}u_{i,y}\right)] \quad (11)$$

The vertices $\vec{u}_i$, $\vec{v}_i$, $\vec{w}_i$ and $\vec{k}_i$ are chosen such that $A_i > 0$.

Let $Q = \{Q_1, Q_2, Q_3, ...\}$ be the set of all quadrilaterals. The energy of the system is

$$U = U_{area} = \sum_{Q_i \in Q} U_i \quad (12)$$

where $U_i$ is the energy of quadrilateral $Q_i$

$$U_i = \frac{1}{2} k_A \left(A_i - A_0\right)^2 \quad (13)$$

such that the quadrilateral energy is a function of the difference between the actual area $A_i$ and the equilibrium area $A_0$.

Subsequently, the gradient is computed for the energy stored in the system:

$$\nabla_n U_{area} = \nabla_n \sum_{Q_i \in \vec{Q}} U_i = \sum_{Q_i \in Q} \nabla_n U_i. \quad (14)$$

The gradient of a single quadrilateral energy term takes the form:

$$\nabla_n U_i = \nabla_n \frac{1}{2} k_A \left(A_i - A_0\right)^2 = k_A \left(A_i - A_0\right) \nabla_n A_i. \quad (15)$$

Note that $\nabla_n U_i$ can be non-zero only if vertex $n$ belongs to $Q_n$, i.e. if $\vec{r}_n$ is one of the vertices $\vec{u}_i$, $\vec{v}_i$, $\vec{w}_i$ or $\vec{k}_i$. To be explicit, let $\vec{r}_n = \vec{u}_i$. In two dimensions $\nabla_n A_i = (\frac{\partial A_i}{\partial u_{i,x}}, \frac{\partial A_i}{\partial u_{i,y}})$. The two components follow immediately from 11:

$$\frac{\partial A_i}{\partial u_{i,x}} = \frac{1}{2}\left(k_{i,y} - v_{i,y}\right),$$

$$\frac{\partial A_i}{\partial u_{i,y}} = \frac{1}{2}\left(v_{i,x} - k_{i,x}\right). \quad (16)$$

Combining equations 9, 15 and 16, the resulting force on vertex $n$ due to the preservation of volume is

$$\vec{F}_n = \sum_{Q_i \ni n} -\frac{1}{2} k_A (A_0 - A_i) \left(k_{i,y} - v_{i,y}, v_{i,x} - k_{i,x}\right). \quad (17)$$

Here the summation is over the quadrilaterals $Q_i$ that contain vertex $n$, and $\vec{k}_i$ and $\vec{v}_i$ are the neighbours of $\vec{r}_n$ in $Q_i$. The vertex will come to rest if $A_i = A_0$, and the strength of the force can be adjusted by parameter $k_A$. All forces are additive.

This was implemented in C++ as a stand-alone application. The program reads in a mesh and evolves the vertices until the system comes to rest.

## Supplementary Figures

Fig S2 shows the results of the simulation in terms of a point spread function. We computed the point spread functions with the prior knowledge that there were six different signals present in the simulation. However, in reality the number of layers that is present in the cortex is often

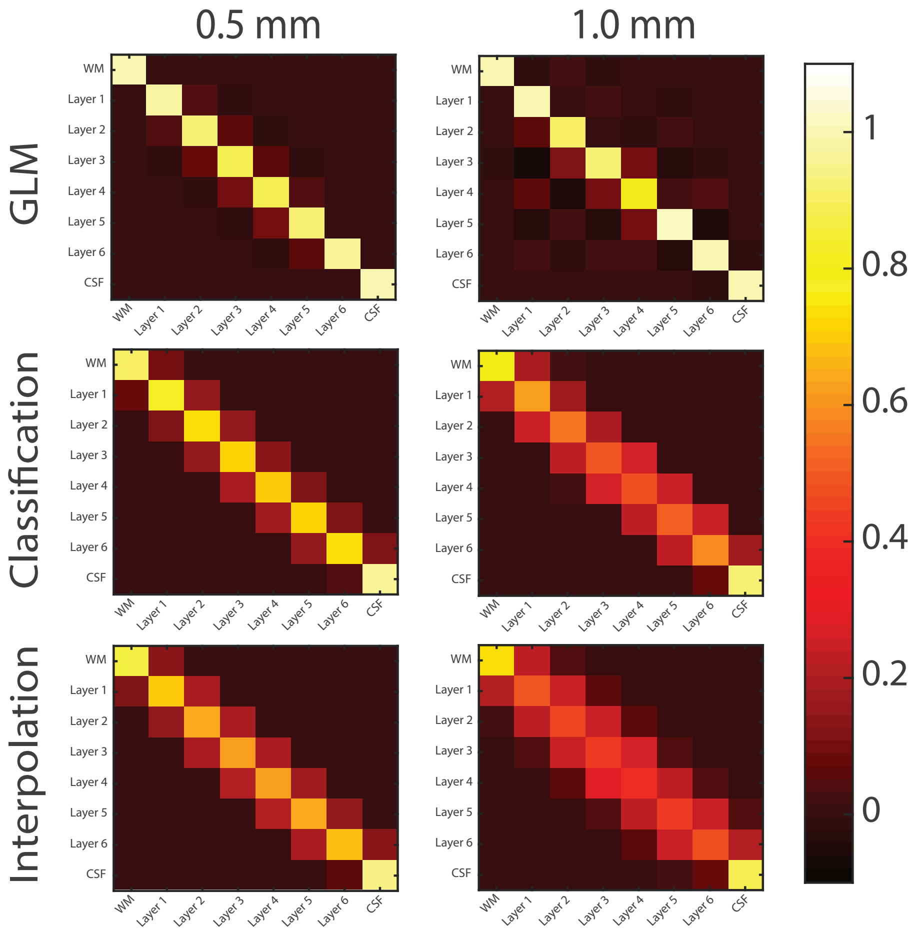


**Fig S2.** The point spread functions of all layers for both resolutions and both methods. An ideal point spread function would look like an identity matrix. This is approached more by the GLM method for both resolutions.

unknown so it is interesting to inspect the profiles when a different number of layers is extracted than is present in the cortex. This is what is shown in Fig S3.

Fig S4 shows the profiles of all subjects individually for all different methods. It is clear that the spatial GLM shows erratic behaviour when larger number of layers are used. Additionally, the interpolation methods shows a smoother profile than the classification approach, indicating that it might be more prone to blurring effects and therefore less specific. For lower numbers of layers, the differences are minimal and, in absence of a gold standard, it cannot be determined which one is objectively better.

# References

1. Bok ST. Der Einfluss der in den Furchen und Windungen auftretenden Krümmungen der Grosshirnrinde auf die Rindenarchitektur. Zeitschrift für die gesamte Neurologie und Psychiatrie. 1929;121(1):682–750.

2. Tallinen T, Chung JY, Biggins JS, Mahadevan L. Gyrification from constrained cortical expansion. Proceedings of the National Academy of Sciences of the United States of America. 2014;111(35):12667–12672.

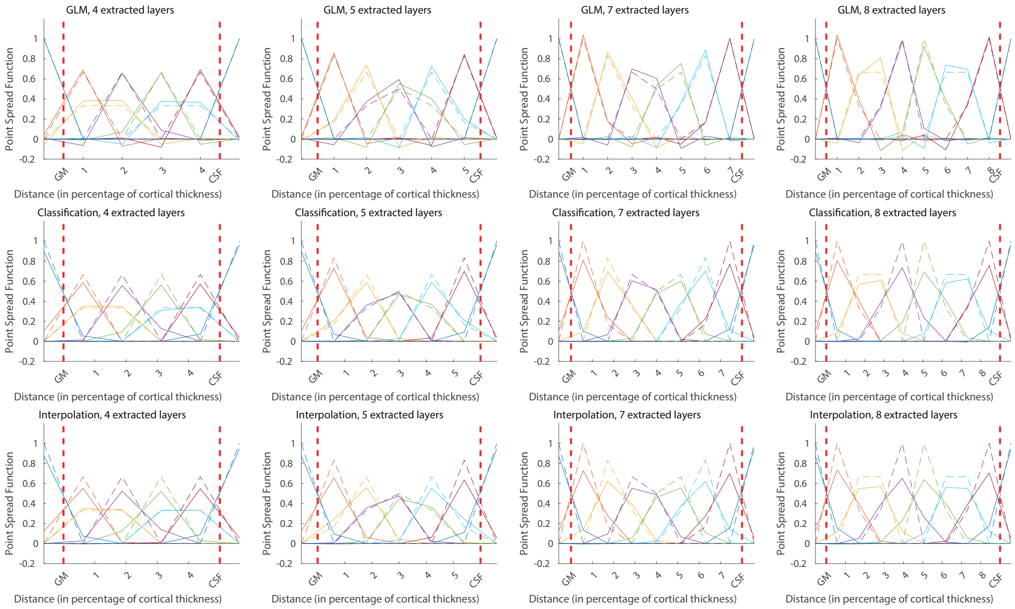


**(a)** The extracted signal with three different methods on the simulated volume of $[0.5 \text{ mm}]^3$

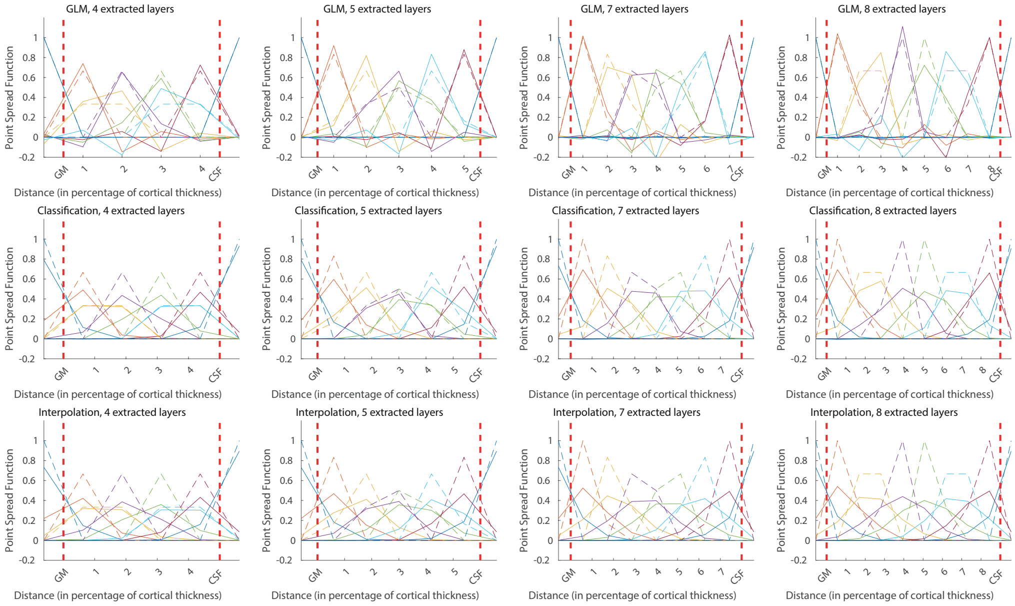


**(b)** The extracted signal with three different methods on the simulated volume of $[1.0 \text{ mm}]^3$

**Fig S3.** This figure shows the extracted signal profiles for the simulation that contains six layers. However, different numbers of layers are extracted (4, 5, 7, 8). Note that the true signal is now spread over different inferred layers, such that the desired outcome is not a set of delta peaks anymore. Instead, the true distribution of the signal is indicated with the dashed lines. The same pattern as for the six-layered cortex emerges: the spatial GLM method is generally closest to the true peak, but is also the only method that shows undershoots in some layers that might induce unwanted anti-correlations.

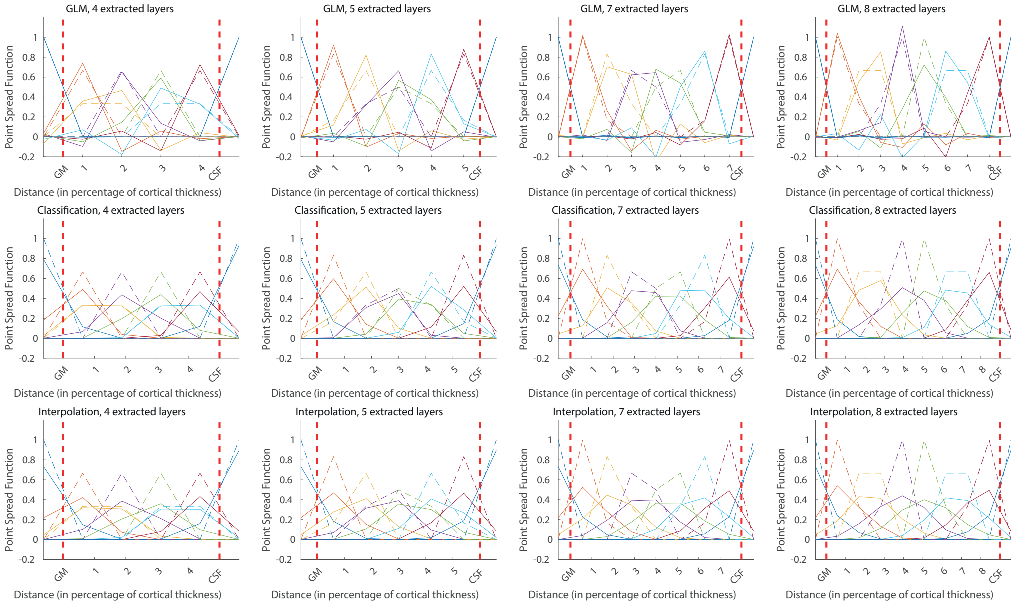


**Fig S4.** The obtained profiles for a small piece of the primary visual cortex, analogous to Figure 5. We here show the profiles for all 11 subjects individually for all methods.
